# Features of breast cancer initially assessed as probably benign on ultrasound: A retrospective study

**DOI:** 10.1097/MD.0000000000029953

**Published:** 2022-08-05

**Authors:** Hye Ji Ryu, Joo Hee Cha, Hak Hee Kim, Hee Jung Shin, Eun Young Chae, Woo Jung Choi

**Affiliations:** a Department of Radiology, Research Institute of Radiology, Asan Medical Center, College of Medicine, University of Ulsan, Seoul, Korea.

**Keywords:** breast neoplasms, missed diagnosis, ultrasonography

## Abstract

We evaluated the features of breast cancers initially assessed as probably benign at ultrasound (US).

Of the 7098 patients who underwent breast cancer surgery at our institution between 2014 and 2016, 179 lesions in 178 patients who had both a prior US with Breast Imaging Reporting and Data System (BI-RADS) category 3 assessment and a recent US with a diagnosis of breast cancer were enrolled. Prior and recent US findings and category were retrospectively reassessed in line with the BI-RADS Atlas and analyzed.

Of the 179 BI-RADS 3 lesions, 105 (59%) were retrospectively reassessed to category 4 and 74 (41%) retained category 3. Noncircumscribed margin, irregular shape, posterior enhancement, and nonparallel orientation were more frequently observed in the reassessment category 4 group than in the reassessment category 3 group (94% vs 43%, 81% vs 19%, 16% vs 4%, 14% vs 0%, respectively). The recent US revealed that 150 of the 179 lesions (84%) had > 20% size increase, and 121 (68%) showed morphologic changes. Margin was the most frequently observed morphologic feature to change (41%, 73/179).

Care should be taken to look for subtle but suspicious US features and changes in mass, especially of margin, for early diagnosis of breast cancer.

## 1. Introduction

Although mammography is the primary imaging method for breast cancer screening, its sensitivity is only 68% to 88% and can decline to as low as 30% to 48% in dense breasts.^[1–4]^ In mammography, breast density, perception error, misinterpretation, and subtle nonspecific findings have been associated with false-negative results.^[5–7]^ A multicenter, retrospective study found that 286 of 427 (67%) breast cancers were retrospectively visible on mammography, but had been initially interpreted as normal.^[5]^

Breast ultrasound (US) is used to evaluate abnormalities detected with mammography, palpable lesions, or screening in women with dense breasts. Previous studies reported that US as supplemental screening increases cancer detection from 1.8 to 4.2 per 1000 women.^[8,9]^ Although US has a higher sensitivity than mammography, 1 major drawback is that it also has high false-positive rate.^[10]^ Nevertheless, breast cancer can still be missed in 22% to 31% on US.^[11,12]^ In US, oval circumscribed hypoechoic mass with parallel orientation, minimal posterior enhancement or no posterior features, complicated cysts, and clustered microcysts are included in category 3 according to BI-RADS Atlas.^[13]^ Because category 3 lesions indicate a likelihood of malignancy of 0% to ≤2%, some of the lesions diagnosed as category 3 are later diagnosed as cancer. Compared with mammography and magnetic resonance imaging, US is a subjective examination that is influenced by the radiologist’s ability and experience and understanding the US features of breast cancer initially assessed as probably benign will help improve diagnostic accuracy. Although many studies have previously focused on the characteristics of breast cancers that have been missed on mammography, few have thoroughly examined the US features of breast cancer initially assessed as probably benign. Therefore, the purpose of our study was to evaluate the features of initially assessed as probably benign on US in women who were subsequently diagnosed with malignancy at follow-up US.

## 2. Methods

This retrospective study was approved by institutional review board of Asan Medical Center (no. 2020-0648) and the requirement for informed consent was waived given the retrospective nature of the study.

### 2.1. Study population

A total of 7097 women with surgically diagnosed breast cancer between January 2014 and December 2016 were identified in our hospital database. Among them, 422 women had received both a prior US and a recent follow-up US that led to a breast cancer diagnosis. In 198 of 422 women, there was a lesion consistent with the lesion later diagnosed as cancer on the prior US. Nineteen women with BI-RADS Category 4 assessment on prior US and 1 woman whose cancer was diagnosed at the mastectomy site were excluded. Finally, 179 lesions in 178 patients (mean age, 47.7 years; age range: 28–71 years) who had prior and recent US in which breast cancer was retrospectively identifiable with BI-RADS category 3 assessment and led to a diagnosis of breast cancer, respectively, were enrolled. Bilateral breast cancer was diagnosed in 1 patient.

Reasons for prior US included follow-up for benign or probably benign lesions (n = 75), screening (n = 67), symptoms (n = 19; 16 palpable lesion, 2 nipple discharge, 1 breast pain), follow-up after breast cancer surgery (n = 15), and abnormal findings on mammography (n = 3). The mean interval between prior and recent US was 9.2 months (range, 6–20 months). Pathologic results of subsequently diagnosed breast cancer were as follows: invasive ductal carcinoma (n = 103), ductal carcinoma in situ (n = 38), microinvasive ductal carcinoma (n = 13), invasive lobular carcinoma (n = 7), mucinous carcinoma (n = 7), tubular carcinoma (n = 3), invasive ductal carcinoma with micropapillary feature (n = 2), tubulolobular carcinoma (n = 2), invasive apocrine carcinoma (n = 2), papillary carcinoma (n = 1), and metaplastic carcinoma (n = 1).

### 2.2. US imaging methods

The US examination was performed by radiologists at our center with experience in breast US (range: 1–22 years) using an IU22 (Philips Medical Systems, Bothell, WA) equipped with a 5 to 12 MHz linear array transducer. Clinical and mammographic information was available before the US.

The breast US scanning technique was standardized across our institution as follows: both breast parenchyma and axilla were included in the examination. With the exception of a simple cyst, 2 images in 2 orthogonal planes (transverse and longitudinal or radial and antiradical planes) with size measurement were obtained in all lesions along with clockwise orientation and distance from the nipple.

### 2.3. Interpretation of US images

First interpretation of prior and recent US images was reported by the examining radiologist. They had access to clinical information and mammography. BI-RADS category of the lesions was recorded according to the American College of Radiology BI-RADS lexicon of US. Doppler US and elastography were performed when examining radiologist thought they were necessary.

Second review of both US images was performed independently by 2 radiologists who specialized in breast imaging (19 and 16 years, respectively) and were not blinded to pathologic information. When there was a disagreement, the 2 radiologists evaluated the case together to achieve consensus. Image features of the lesions, including shape, orientation, margin, echo pattern, posterior feature, calcification on prior and recent US, and BI-RADS category of the lesion on the prior US, were reassessed according to the American College of Radiology BI-RADS lexicon of US. Doppler US and elastography were not evaluated in second review. As previously described, multiplicity of the lesion on the prior US was recorded and defined when there were at least 3 circumscribed masses, with 1 in each breast.^[14]^ Maximum diameter of the lesions on the prior and recent US was recorded. Mean size change, mean size change per month, mean percentage change ([maximum diameter on recent US—maximum diameter on the prior US] / maximum diameter on prior US × 100), and mean percentage change per month were calculated.^[15]^ The number of cases to show a morphologic change or >20% increase in maximum diameter over 6 months was also evaluated.^[16]^

### 2.4. Data and statistical analysis

The lesions were divided into 2 groups, those that received reassessment category 3 and 4 on second review. Imaging and clinical features of reassessment category 3 and 4 lesions were compared using the *t* test for continuous variables, and chi-square or Fisher exact test for categorical variables. Bonferroni correction was used for multiple comparisons. Statistical analysis was performed using SPSS, version 18.0 (SPSS Inc., Chicago, IL), and *P* values of <.05 were considered statistically significant.

## 3. Results

### 3.1. US features of breast cancer initially assessed as probably benign

Of the 179 lesions initially categorized as BI-RADS 3, 105 lesions (59%) were reassessed to category 4, while 74 lesions (41%) were retained as category 3.

**Figure 1.**
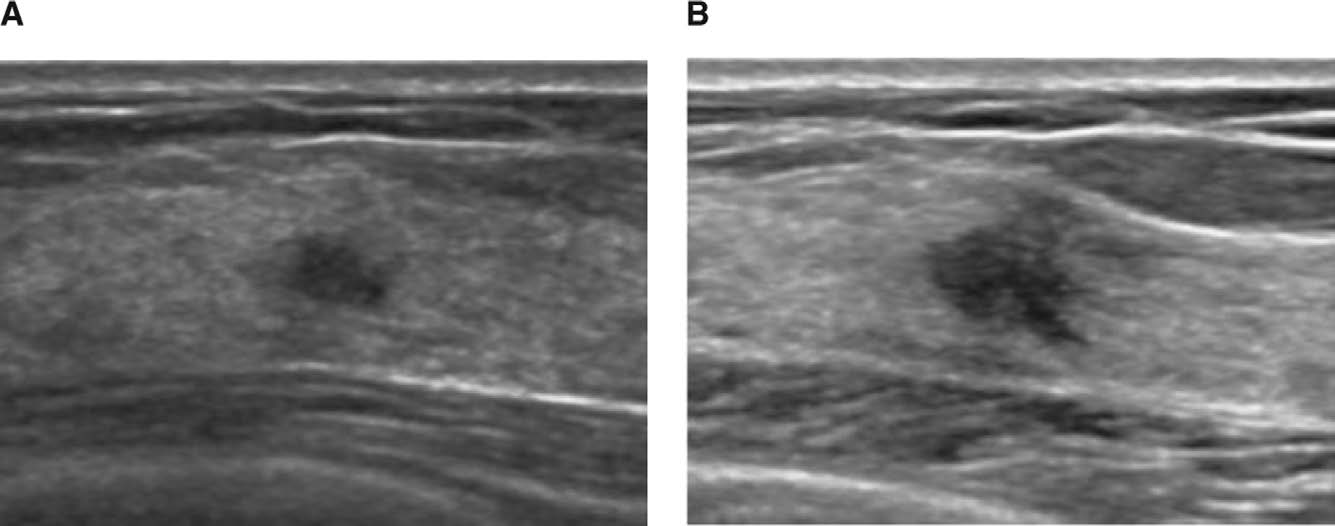
Screening US of a 50-yr-old woman. (A) Prior US shows 0.5 cm sized hypoechoic mass with an irregular shape and indistinct margin. This mass was initially assessed as BI-RADS category 3 but upgraded to BI-RADS category 4 in reassessment due to its shape and margin. (B) A 7-mo follow-up US reveals a size increment of the mass to 0.8 cm. This mass was confirmed as invasive ductal carcinoma after surgery. BI-RADS = Breast Imaging Reporting and Data System, US = ultrasound.

Age and multiplicity of lesions were not significantly different between reassessment category 3 and 4 groups (47.8 ± 9.0 vs 47.6 ± 9.3, *P* = .91; 53% vs 48%, *P* = .50). The mean lesion size on the prior US in the reassessment category 4 group was larger than that in the reassessment 3 group (10.3 ± 6.2 mm vs 6.7 ± 2.8 mm, *P* < .001). Noncircumscribed margin, irregular shape, posterior enhancement, and nonparallel orientation were more frequently observed in the reassessment category 4 group compared with the reassessment category 3 group (94% vs 43%, *P* < .001; 81% vs 19%, *P* < .001; 16% vs 4%, *P* = .007; 14% vs 0%, *P* = .001, respectively; Table 1, Fig. 1). Among the noncircumscribed margins, indistinct and microlobulated margins were frequently ignored, in 51/99 and 36/99 lesions, respectively. There was no significant difference in echo pattern (*P* = .60) and presence of calcification (*P* = .40).

**Table 1:** US features of breast cancer initially assessed as probably benign.

	Total (n = 179)	Reassessment category		*P* value
		3 (n = 74)	4 (n = 105)	
Age, yr	47.7 ± 9.2	47.8 ± 9.0	47.6 ± 9.3	.91
Multiplicity, n (%)	89 (50)	39 (53)	50 (48)	.50
Size, mm	8.8 ± 5.3	6.7 ± 2.8	10.3 ± 6.2	<.001
Shape, n (%)				<.001
Oval, round	80 (45)	60 (81)	20 (19)	
Irregular	99 (55)	14 (19)	85 (81)	
Orientation, n (%)				.001
Parallel	164 (92)	74 (100)	90 (86)	
Nonparallel	15 (8)	0	15 (14)	
Margin, n (%)				<.001
Circumscribed	48 (27)	42 (57)	6 (6)	
Noncircumscribed	131 (73)	32 (43)	99 (94)	
Indistinct	64 (36)	13 (18)	51 (49)	
Angular	12 (7)	1 (1)	11 (10)	
Microlobulated	54 (30)	18 (24)	36 (34)	
Spiculated	1 (1)	0	1 (1)	
Echo pattern, n (%)				.60
Hyperechoic	1 (1)	0	1 (1)	
Complex cystic and solid	6 (3)	1 (1)	5 (5)	
Hypoechoic	167 (93)	72 (97)	95 (90)	
Isoechoic	1 (1)	0	1 (1)	
Heterogeneous	4 (2)	1 (1)	3 (3)	
Posterior features, n (%)				.001*
No posterior features	152 (85)	71 (96)	81 (77)	
Enhancement	20 (11)	3 (4)	17 (16)	
Shadowing	7 (4)	0	7 (7)	
Combined pattern	0	0	0	
Calcification, n (%)				.40
Absent	173 (97)	73 (99)	100 (95)	
Present	6 (3)	1 (1)	5 (5)	

### 3.2. Interval change of breast cancer initially assessed as probably benign

**Figure 2.**
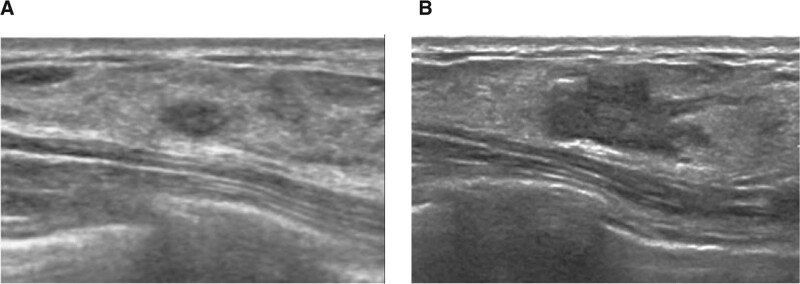
Screening US of 32-yr old woman with a family history of breast cancer. (A) Prior US showed 0.6 cm sized oval and circumscribed hypoechoic mass. The mass was categorized as BI-RADS category 3 in both initial and reassessment. (B) On follow-up US after 14 mo, the mass had increased to 1.9 cm with a morphologic change to irregular shape and angular margin. This mass was confirmed as ductal carcinoma in situ. BI-RADS = Breast Imaging Reporting and Data System, US = ultrasound.

**Figure 3.**
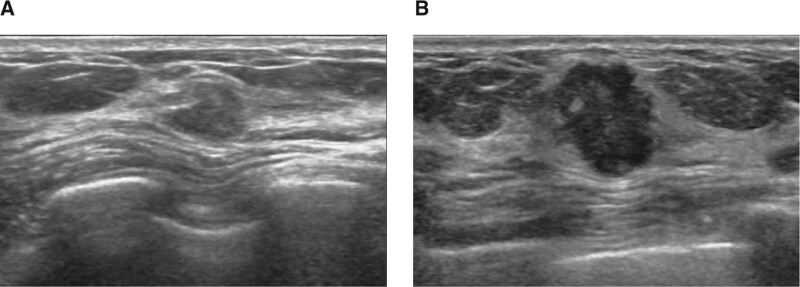
Diagnostic US for mammographic abnormality of a 68-yr-old woman. (A) Prior US shows 0.7 cm sized hypoechoic mass with an irregular shape and microlobulated margin. This mass was initially assessed as BI-RADS category 3 but reassessed as BI-RADS category 4 on the second review due to its shape and margin. (B) An 8-mo follow-up US revealed size increment to 1.2 cm, change in orientation from parallel to nonparallel, and newly developed posterior enhancement observed. This mass was confirmed as invasive apocrine carcinoma. BI-RADS = Breast Imaging Reporting and Data System, US = ultrasound.

The interval change of subsequently diagnosed breast cancer between prior and recent US is shown in Table 2. Of the 179 lesions, 150 (84%) showed ≥20% size increase, and 121 (68%) showed morphologic changes. One hundred eight of the 179 lesions (60%) showed ≥20% size increase and morphologic change (Figs. 2 and 3). Mean size change of maximal diameter and mean size change of maximal diameter per month of the lesions between prior and recent US were 7 ± 5 and 1 ± 0.9 mm, respectively. The mean percentage change of maximal diameter and mean percentage change of maximal diameter per month were 134.5 ± 104.8 and 20.3 ± 19.2, respectively. Lesion margin was the most frequently observed feature to change (41%, 73/179), followed by shape (24%, 43/179), and posterior feature (24%, 43/179). Twenty-one of 73 (29%) lesions evolved from circumscribed margin to microlobulated margin, and 13 of 73 lesions (18%) evolved from circumscribed to indistinct margin. Concerning shape, all lesions except 1 (42/43, 98%) transitioned from an oval or round shape to an irregular shape. Of the 43 lesions with the posterior feature change, posterior enhancement had newly developed in 27 lesions (63%), and posterior shadowing had developed in 12 lesions (28%). Sixteen of the 179 lesions (9%) did not show a significant size increase nor morphologic change at second review. Although these 16 lesions without significant interval change were initially assessed as category 3 on the prior US, this was updated to category 3 (n = 1) and category 4 (n = 15) on the second review of the prior US. The recent US had assessed these lesions as category 3 (n = 1), category 4 (n = 14), and category 5 (n = 1). A biopsy on the lesion that was classified as category 3 on both second review of prior US and recent US was performed at the patient’s request, and ductal carcinoma in situ was confirmed on surgical excision.

**Table 2:** Interval change of breast cancer initially assessed as probably benign.

	Total (n = 179)
Interval between prior and recent US, mo	9.2 ± 3
Size change, mm	7 ± 5
Size change per month, mm	1 ± 0.9
Size change, %	134.5 ± 104.8
Size change per month, %	20.3 ± .19.2
Size change, n (%)	
≤20%	29 (16)
>20%	150 (84)
Morphologic change, n (%)	
Yes	121 (68)
Type	2 (1)
Shape	43 (24)
Orientation	18 (10)
Margin	73 (41)
Echo pattern	9 (5)
Posterior feature	43 (24)
Calcification	8 (4)
No	58 (32)
Size (>20%) and morphologic change, n (%)	108 (60)
No change in size (>20%) and morphology, n (%)	16 (9)
Category on second review of prior US, n (%)	
3	1 (6)
4	15 (94)
Category on recent US, n (%)	
3	1 (6)
4	14 (88)
5	1 (6)

## 4. Discussion

On our second review of 179 lesions initially assessed as probably benign and subsequently diagnosed as breast cancer, 59% (105/179) were reassessed as BI-RADS category 4, suggesting that they could have been diagnosed at the time of the prior US. Two previous studies reported that 79% (52/72) and 91% (29/32) of visible lesions on the initial US, subsequently diagnosed as malignant, were actionable.^[12,17]^ They also reported that suspicious margin characteristics were the most common missed features, as observed in our study. In previous studies, the positive predictive value of indistinct, angular, microlobulated, and spiculated margin were reported as 46% to 67%, 60% to 91%, 60% to 100%, and 86% to 88%, respectively.^[18,19]^ Despite the high positive predictive value of noncircumscribed margin, this finding is sometimes overlooked in clinical practice, especially if the lesion is small. This is because it is also important to reduce unnecessary biopsies. In our study, the mean size of the lesions on prior US was greater in the reassessment category 4 group than in the reassessment category 3 group (10.3 ± 6.2 vs 6.7 ± 2.8 mm, *P* < .001). Tendency to ignore suspicious features, particularly in small lesions, may have affected second review of prior US. In addition, Abdullah et al^[20]^ reported that interobserver agreement of margin was lower than that of shape, orientation, echo pattern, and posterior features. They also reported lower reader agreement of margin in malignant masses compared to benign masses. Given these previous studies and our result, when evaluating lesions on US, attention should be paid to the margin, shape, posterior feature, and orientation of the lesions (especially margin) to decrease false-negative assessment. Also, with efforts to reduce false-negative assessment, the risk that false-positive assessment may increase should be considered.

Any lesion with suspicious morphological change or with a size increase of >20% within 6 months should be categorized as BI-RADS 4 and recommended for biopsy.^[13,16]^ The malignancy rates of probably benign lesions with interval change on follow-up US range from 4.9% to 10.3%.^[15,21,22]^ Previous studies have reported that mean diameter change per month was greater in malignant lesions compared with benign lesions (0.6 vs 0.4 mm [8.6% vs 4.9%], 1.8 vs 0.5 mm), and the malignancy rate of lesions with morphologic change was higher than lesions without (13.6% vs 4.9%, 38.5% vs 4%).^[15,22]^ In our study, 84% (150/179) of lesions initially categorized as 3 and subsequently diagnosed as malignant showed a ≥ 20% interval size change and mean size change per month was 1 mm (20%). Sixty-eight percent (121/179) of these lesions showed morphologic change. Margin (41%, 73/179) was the feature that changed most often, followed by shape (24%, 43/179), and posterior feature (24%, 43/179), similar to missed features on the prior US. This is comparable with previous study that reported highest rate of change of margin (77.8%, 7/9) followed by shape (44.4%, 4/9) in malignant lesions that showed morphologic change.^[15]^ So as mentioned earlier, the margin should be carefully evaluated not only in the initial evaluation of mass but also in follow-up US. Interestingly, posterior enhancement developed in 27 of 179 lesions at follow-up US. Although the cause of posterior enhancement in breast malignancy is unclear, a previous study has suggested it may be attributed to the tumor tissue’s high cellularity and organization.^[23]^ It is important to consider the 16 lesions later diagnosed as malignant without significant size (>20%) or morphological changes on the second review of the prior US and recent US. These lesions were initially assessed as category 3 on the prior US; however, all but 1 were later upgraded upon reassessment of prior US and recent US. This suggests that lesions with any suspicious features should be examined meticulously, even if major changes have not been observed since the prior US; there remains the possibility of false-negative assessment during the prior US. Not only interval change, but thorough examination of suspicious features is required on follow-up US.

Our study has several limitations. First, this was a retrospective study performed at a single institution with a small sample size. Second, we cannot exclude the cancers which have naturally progressed from benign lesions to cancers. Actually, of 179 lesions that were newly assessed as category 4 on recent US, 74 lesions had the benign features on the previous US, even with second review. However, others would have been diagnosed as BI-RADS 4 if they had been more carefully evaluated for their subtle but suspicious US features on the previous US. We focused on these subtle but suspicious findings and it will help reduce delays in breast cancer diagnosis. Third, our reviewers evaluated the images with the awareness of the final pathology, and this might have influenced the assessment of lesions. Two specialized radiologists reviewed image with a consensus, to reduce the effect. Fourth, lesions were assessed only on static B-mode US images because of the retrospective nature of our study. Because most of the lesions were not evaluated with color Doppler and elastography images at the time of real-time US due to their benign appearance, these images were not evaluated on the second review.

In conclusion, upon second review of the prior US, 59% of lesions initially assessed as probably benign lesions but subsequently diagnosed as “malignant” were deemed suspicious. Understanding the features of breast cancer that allows them to go diagnostic delay is essential to reducing their prevalence and improving patient outcomes. For early detection of breast cancers, care should be taken to look for subtle but suspicious US features and changes in masses, especially of lesion margin.

## Footnotes

* No posterior feature vs enhancement, *P* = .007; no posterior feature vs shadowing, *P* = .017, enhancement vs shadowing, *P* = .55 (significant values α = 0.05/3 = 0.017).
